# High positive predictive value of the combined pituitary dynamic enhanced MRI and high-dose dexamethasone suppression tests in the diagnosis of Cushing’s disease bypassing bilateral inferior petrosal sinus sampling

**DOI:** 10.1038/s41598-020-71628-0

**Published:** 2020-09-07

**Authors:** Zeyu Liu, Xiaobo Zhang, Zhiwei Wang, Hui You, Mingli Li, Feng Feng, Zhengyu Jin

**Affiliations:** Department of Radiology, Peking Union Medical College Hospital, Peking Union Medical College, Chinese Academe of Medical Sciences, Beijing, 100730 China

**Keywords:** Pituitary diseases, CNS cancer

## Abstract

The purpose of the study is to evaluate the positive predictive value of the combined pituitary dynamic enhanced MRI (dMRI) and high-dose dexamethasone suppression tests (HDDST) in the diagnosis of Cushing’s disease (CD) bypassing bilateral inferior petrosal sinus sampling (BIPSS). A total of 118 patients with Cushing’s syndrome (CS), who underwent pituitary dMRI, HDDST and BIPSS were included. Positive predictive value of pituitary dMRI, HDDST, BIPSS and the combined test were calculated and tumor lateralization accuracy was further analyzed. The positive predictive value of the combined pituitary dMRI and HDDST was 98.6%, higher than that of BIPSS. There were 96.8% of patients, who had either negative findings in pituitary dMRI or HDDST, showing centralizing BIPSS results. For tumor lateralization, the accuracy by pituitary dMRI was 88.6%, whereas BIPSS was 57.5%. Therefore, CS patients with both positive findings in pituitary dMRI and HDDST need no further invasive evaluation to establish the definite diagnosis of CD. BIPSS will improve the diagnostic accuracy when negative findings were found in either pituitary dMRI or HDDST.

## Introduction

Cushing’s syndrome (CS) is a disorder with a cluster of clinical manifestations, such as central obesity, moon facies, purple striae and hypertension, which is mostly attributed to excess endogenous or exogenous glucocorticoid. Endogenous CS can be classified into adrenocorticotropic hormone (ACTH)-dependent and ACTH-independent CS. Cushing’s disease (CD), the excess ACTH secretion of pituitary adenomas, is the most common etiology of ACTH-dependent CS. Other causes of ACTH-dependent CS include ectopic ACTH and ectopic corticotropin-releasing hormone (CRH) secretion, which are known as ectopic ACTH syndrome (EAS) and ectopic CRH syndrome, respectively^1,2^. The differential diagnosis of ACTH-dependent CS is important and it will decide a specific treatment.

There are considerable challenges in the identification of CD. Pituitary MRI, high-dose dexamethasone suppression tests (HDDST) and bilateral inferior petrosal sinus sampling (BIPSS) are widely used in the diagnosis of CD and their diagnostic performance have been intensively investigated in previous studies. In the past, it was reported that up to 50% of patients had false-negative results in pituitary MRI^3^. Given the false-positive results caused by nonfunctioning pituitary adenomas, positive findings in pituitary MRI alone can not confirm the diagnosis of CD. The limited diagnostic value impedes the establishment of CD diagnosis. BIPSS shows high diagnostic accuracy, the sensitivity and specificity of which were 90% and 90%-95%, respectively^4–7^. Although BIPSS is an invasive and high-technical procedure, some authorities suggest that BIPSS should be performed in all the patients with ACTH-dependent CS^1^. On the contrary, other authorities hold the opinion that BIPSS can be safely bypassed in some ACTH-dependent CS patients^7^.

As the development of MRI technology, such as pituitary dynamic enhanced MRI (dMRI) and high-resolution MRI, the detection rate of pituitary adenomas has been significantly improved^8^. Compared with conventional MRI, dMRI provides more information about the pituitary adenoma with repeated images over a few seconds after contrast injection, which potentially help improve the diagnosis and the treatment. Therefore, the aim of the current study is to explore whether positive findings in both pituitary dMRI and HDDST are sufficient to establish the diagnosis of CD, where BIPSS can be safely bypassed. Moreover, the effects of tumor lateralization in the invasive and non-invasive evaluation will be investigated in the diagnosis of CD.

## Methods

### Subjects

We retrospectively reviewed the medical records and imaging studies of all the ACTH-dependent CS patients presented to Peking Union Medical College Hospital from January 2015 to December 2018. Patients who underwent pituitary dMRI, HDDST and BIPSS were included in the study. Patients who underwent conventional MRI instead of pituitary dMRI, or lack of HDDST or BIPSS results were excluded from the retrospective study. Finally, 118 patients were included in the current study. The diagnosis of CS was based on the criteria in the Endocrine Society Clinical Practice Guideline^9^. The confirmation was made by a pathologically proven adenoma or by clinical remission after surgery. Patient information was obtained with their permission and informed consent was obtained from the subjects.

### HDDST

Before HDDST, 2 days of 24-h urinary free cortisol (24hUFC) were measured. The average 24hUFC level was recorded as baseline. In the HDDST, 2 mg dexamethasone was administered orally every 6 h for 2 days and the 24hUFC level of the second day of HDDST was measured. It was considered to be suppressed in the HDDST that the ratio of 24hUFC after HDDST to 24hUFC at baseline was less than 50%. The suppression in the HDDST, considered to be consistent with CD, was marked as positive in the current study. Patients suspected cyclic CS were tested at the active phase instead of the rest phase to avoid false-negative results.

### Imaging

Pituitary dMRI was performed in all the patients on a 3.0 T MR scanner (Discovery MR750, GE Healthcare). Coronal T1-weighted imaging was acquired with fast spin echo (FSE, TR = 417 ms, TE = 8.9 ms, echo train = 3, FOV = 20 cm × 16 cm, matrix = 320 × 224, slice thickness = 3 mm, gap = 0.6 mm). Sagittal T1-weighted imaging was acquired with fat-saturated CUBE (TR = 300 ms, TE = 15.6 ms, echo train = 9, FOV = 17 cm × 17 cm, matrix = 256 × 192, slice thickness = 3 mm, gap = 0 mm). Coronal T2-weighted imaging was performed with fast recovery fast spin echo (FRFSE, TR = 3,898 ms, TE = 89.8 ms, echo train = 20, FOV = 20 cm × 16 cm, matrix = 320 × 224, slice thickness = 4 mm, gap = 1 mm). Coronal FSE T1 dynamic post-gadolinium (TR = 300 ms, TE = 16 ms, echo train = 3, FOV = 19 cm × 16 cm, matrix = 224 × 192, slice thickness = 2 mm, gap = 0.5 mm, 21 s per phase with 6-phase) was performed during the intravenous gadolinium administration (0.05 mmol/kg), and then coronal and sagittal T1-weighted imaging were repeated.

It was suspected as a pituitary adenoma that a discrete hypointensity in early phase of dMRI becoming less hypointense with subsequent dynamic cycles. The localization and the size of the lesion were recorded. All the images were independently reviewed by two experienced neuroradiologists blinded to the clinical information with 16 and 26 years of experience and the final decision was made through consensus agreement.

### BIPSS

BIPSS was performed as described by Doppman et al. by two experienced interventional radiologists, with 11 and 23 years of experience^10^. Bilateral femoral veins were accessed without anticoagulation and four French catheters were guided into bilateral inferior petrosal sinuses (IPSs), where patients with narrow IPSs used microcatheters instead. Retrograde venography was utilized for the confirmation of catheters’ position. Before the desmopressin stimulation test, blood samples were collected from peripheral veins and bilateral IPSs. After the introduction of 10 μg desmopressin, blood samples were collected at baseline and multiple time points (3, 5 and 10 min). All the blood samples were immediately delivered for ACTH assay. According to previously established criteria, an IPS to peripheral ACTH ratio of ≥ 2.0 in the basal state or ≥ 3.0 after desmopressin stimulation at any time point is consistent with CD^11^. In the current study, the centralizing BIPSS was marked as positive, suggesting the pituitary sources of ACTH. Tumor lateralization was furthermore predicted by an intersinus ratio of ≥ 1.4^11^.

### Treatment and pathology

Transsphenoidal surgery was conducted in patients with the diagnosis of CD and tumor lateralization was recorded. A total pituitary exploration was performed if no tumor was visualized. Resection of the ectopic ACTH-secreting tumor was performed in patients with EAS. The diagnosis was confirmed by pathologically positive immunohistological staining for ACTH or by clinical remission after surgery, whose postoperative serum cortisol was within or below the normal range.

### Statistical analysis

Statistical analysis was completed using SPSS Statistics 22 software. Continuous variables were interpreted by mean ± standard deviation or median (first quartile, third quartile) and were analyzed using the Student *t* test or Mann–Whitney *U* test. Categorical variables were determined using Chi-square test or Fisher’s exact test. P value less than 0.05 was considered statistically significant.

### Ethics statement

The study was approved by the Institutional Review Board of Peking Union Medical College Hospital, Chinese Academy of Medical Sciences, and conducted in accordance with the Helsinki Declaration.

## Results

### Baseline characteristics

A total of 118 ACTH-dependent CS patients who underwent pituitary dMRI, HDDST and BIPSS were included in the study. There were 102 (86.4%) CD patients and 16 (13.6%) EAS patients. Of the 118 CS patients, 86 (72.9%) were female and 32 (27.1%) were male. The average age of the patients was 38.5 ± 14.2 years (range 11–76 years), with 12 (10.2%) children included. The median duration of the disease was 4 (1, 6) years. The median ACTH level was 77.7 (50.8, 113.0) ng/L (normal range 0–46 ng/L) and 24hUFC level was 472.6 (236.0, 820.3) μg (normal range 34–286 μg).

**Figure 1:**
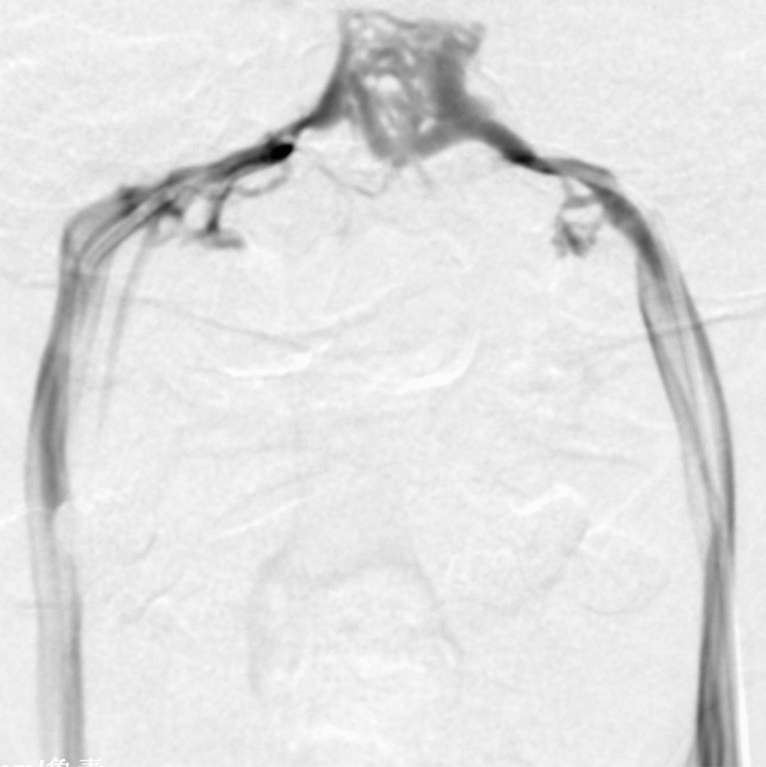
The angiography of bilateral inferior petrosal sinus. Catheters were successfully guided into bilateral inferior petrosal sinus in a 35-year-old patient.

All the patients underwent pituitary dMRI and HDDST with standard procedure, and no side effects were reported. BIPSS were technically successful in all the cases in the current study (Fig. 1). Microcatheters were used in 83 (70.3%) patients with narrow IPSs. No complications were found except 3 (2.5%) patients with groin hematoma in BIPSS procedures, which resolved without further treatment.

### Combined pituitary dMRI and HDDST versus BIPSS

Detailed diagnostic information of the tests was demonstrated in Table 1. There were 72 patients having positive findings in both pituitary dMRI and HDDST, of whom 71 patients were confirmed with CD. Hence, the positive predictive value of the combined test was 98.6%. The combined test carried an acceptable sensitivity of 69.6%.

**Table 1:** Positive predictive value of different tests in 118 patients.

	Diagnosis		Positive predictive value (%)
	CD	EAS	
**Pituitary dMRI**			93.3
Positive	83	6	
Negative	19	10	
**HDDST**			93.5
Positive	86	6	
Negative	16	10	
**BIPSS**			97.0
Positive	97	3	
Negative	5	13	
**Combined pituitary dMRI and HDDST**			98.6
Both positive	71	1	
At least one negative	31	15	

*CD* Cushing’s disease, *EAS* ectopic adrenocorticotropic hormone syndrome, *dMRI* dynamic enhanced MRI, *HDDST* high-dose dexamethasone suppression tests, *BIPSS* bilateral inferior petrosal sinus sampling.

Further, the analysis was conducted in the 46 patients who had either negative findings in pituitary dMRI or HDDST, of whom 15 patients were EAS and 31 were CD. Among the 31 CD patients, 30 (96.8%) patients had centralizing BIPSS results, which were consistent with CD.

According to the findings in combined pituitary dMRI and HDDST, subgroup analysis was conducted within the 102 CD patients. No significant difference was found between true positive group and false negative group, including gender, age, ACTH level, 24hUFC level, and diameter of the tumor.

### Tumor lateralization accuracy

**Figure 2:**
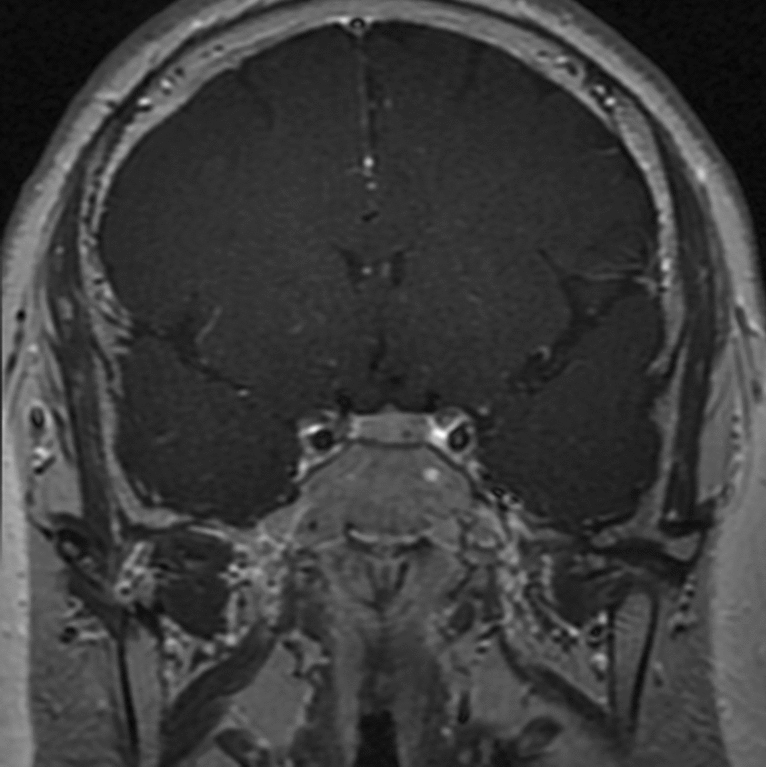
Pituitary dynamic enhanced MRI for pituitary adenomas. A 3 mm pituitary adenoma was shown in the left sella with delayed enhancement.

Tumor lateralization was recorded in pituitary dMRI as well as BIPSS. No tumor in pituitary dMRI was found in 19 of the 102 CD patients. There were 83 patients with positive findings in pituitary dMRI, the average diameter of whose tumor was 6.2 ± 3.4 mm. According to pituitary dMRI, 72 (86.7%) patients had microadenomas defined as tumors no greater than 10 mm at the largest dimension (Fig. 2).

Among the 71 CD patients who had positive findings in both pituitary dMRI and HDDST, 1 (1.4%) patient underwent a total pituitary exploration because of no visible tumor in surgery, 37 (52.1%) were right-sided of the pituitary gland in surgery, 27 (38.0%) were left-sided and 6 (8.5%) were in the middle. As shown in Table 2, there were 62 patients whose tumor lateralization in pituitary dMRI were concordant with surgery. Eight patients lateralized to contralateral side. Hence, the accuracy in lateralization by pituitary dMRI was 88.6%.

**Table 2:** Tumor lateralization by pituitary dMRI and surgery.

	By surgery		
	Left	Right	Middle
**By pituitary dMRI**			
Left	21	2	0
Right	6	35	0
Middle	0	0	6

*dMRI* dynamic enhanced MRI.

Among the 97 CD patients with positive BIPSS findings, 40 (41.2%) patients had BIPSS lateralization suggested by an intersinus ratio of ≥ 1.4^11^. As indicated in Table 3, there were 23 patients had BIPSS lateralization that were concordant in laterality wit surgery. Thus, the lateralization accuracy of BIPSS was 57.5%.

**Table 3:** Tumor lateralization by BIPSS and surgery.

	By surgery	
	Left	Right
**By BIPSS**		
Left	10	4
Right	13	13

*BIPSS* bilateral inferior petrosal sinus sampling.

## Discussion

The differential diagnosis of CS is considerably challenging. In the current study, we investigated the invasive and non-invasive tests in the identification of CD among patients with CS. It was showed that the positive predictive value of the combined pituitary dMRI and HDDST was 98.6%, which is higher than that of BIPSS.

**Figure 3:**
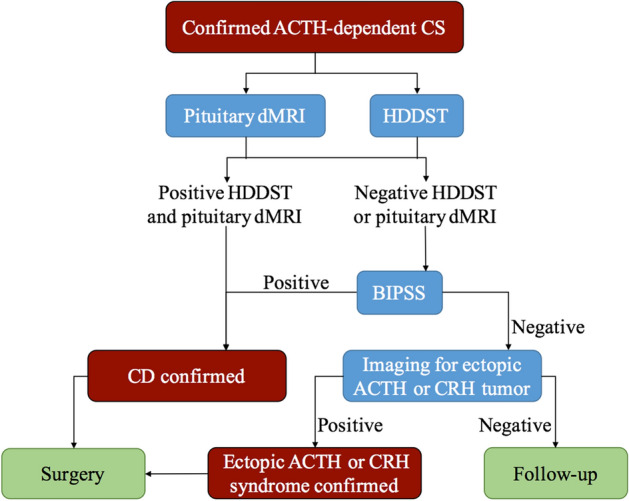
The flowchart for the differential diagnosis of confirmed adrenocorticotropic hormone-dependent Cushing’s syndrome. *ACTH* adrenocorticotropic hormone, *CS* Cushing’s syndrome, *dMRI* dynamic enhanced MRI, *HDDST* high-dose dexamethasone suppression tests, *BIPSS* bilateral inferior petrosal sinus sampling, *CD* Cushing’s disease, *CRH* corticotropin-releasing hormone.

It is still a controversial topic whether BIPSS should be performed in all the patients with ACTH-dependent CS. The attitude towards this topic lead to different clinical decision-making process. Some physicians prefer to start with non-invasive procedures instead of BIPSS^2^. BIPSS will be bypassed in patients with concordant results in HDDST and pituitary MRI^2,7^. According to the opinion of the consensus statement, some patients need no further invasive evaluation to establish the definite diagnosis, whose pituitary lesion was greater than 6 mm in pituitary MRI and had a classical presentation and dynamic biochemical results consistent with CD^7^. On the contrary, BIPSS will be scheduled once the diagnosis of ACTH-dependent CS is established^1^. Considering that 10% to 40% of the population harbor nonfunctioning pituitary adenomas^7,12^, pituitary MRI will be processed after centralizing BIPSS results were found^1^. Another possible reason to perform BIPSS is the false-negative results in conventional pituitary MRI, of which the rate was reported up to 50% in the past^3^. Bekci et al. found that the sensitivity of MRI was only 56%, whereas the sensitivity of BIPSS before and after CRH stimulation was 93.3% and 100%, respectively^13^. However, pituitary dMRI improves the detection of pituitary adenomas compared with conventional MRI^8,14^. In addition to the high positive predictive value, the combined pituitary dMRI and HDDST has a moderate sensitivity, which help 69.6% of CD patients bypass BIPSS in our study (Fig. 3).

Tumor lateralization in CD patients is concerned because resection of primary lesion is recommended as the first-line treatment^15^. The accuracy of lateralization by BIPSS was widely explored, ranging from 48.0 to 78.7%^16–18^. There were 57.5% of the patients having concordant lateralization in our study, which is similar to previous studies. The normal symmetric IPSs help improve the BIPSS lateralization. However, considerable variations of the anatomy of IPSs limit the accuracy, such as discordance in lateralization of the tumor and the dominant venous drainage. Many factors contribute to the low accuracy of BIPSS lateralization in CD patients, of which the venous drainage pathways of the petrosal sinus may be the dominant influence^19^. The inaccuracy will lead to a total pituitary exploration. Although the sensitivity of pituitary MRI is lower than BIPSS in the detection of CD, the lateralization by pituitary MRI is more accurate, especially by pituitary dMRI^14,16^. The accuracy in lateralization by pituitary dMRI (88.6%) was higher than that of BIPSS (57.5%). Therefore, pituitary dMRI is more helpful in tumor lateralization to assist surgeons in transsphenoidal surgery.

The cost-effectiveness of the evaluation should be taken into consideration, especially in the developing countries. The sensitivity of BIPSS has been increased since Landolt improved the BIPSS with CRH stimulation based on Corrigan’s method^20,21^. But CRH is expensive and has limited availability. Desmopressin, which is cheap and easily available, is always used as a substitute of CRH with comparable effects^22,23^. Even so, the BIPSS procedure itself is a high-cost procedure, which will be more expensive if microcatheters are applied in patients with narrow IPSs. Further, technical failure and adverse events of BIPSS should be noticed as an invasive procedure. The technical success rate in our hospital is high and no severe complication occurs. However, there are no more than ten academic medical centers in China that are comparable to our hospital, which can not afford the need of the patients. Compared with BIPSS, pituitary dMRI and HDDST are cheaper and much safer in terms of their non-invasive merits. In the current study, the diagnosis can be made in 69.6% of CD patients after the combined test without BIPSS, which help reduce the cost and the risk of the invasive evaluation. Additionally, pituitary dMRI and HDDST are easier to operate. False negative findings in BIPSS due to technical reasons may mislead the diagnosis, lengthening the diagnosis process. The disease duration in the current study was about 4 years. Early diagnosis is conducive to timely treatment of patients.

In addition, the prevalence of CS is low, making it difficult to include a large number of patients for investigation. To our knowledge, previous studies took decades to include over 100 patients, during which time changes could occur in the MRI parameters or the biochemical measurement. In our study, the relative short time span minimized the potential interference due to these factors, which made the results in our study convincible and reliable that not all the ACTH-dependent CS patients deserve BIPSS (Fig. 3). Considering that the new MRI technology allows the detection of smaller pituitary adenomas than conventional MRI, the “6 mm” threshold put forward by the consensus statement should be reconsidered in future studies (Fig. 2).

Limitations of the current study include its retrospective nature. The bias may be introduced in regard to which patients underwent all the pituitary dMRI, HDDST, BIPSS and had pathological confirmation or clinical remission after treatment. In fact, ACTH-dependent CS patients with concordant results in HDDST and pituitary MRI were diagnosed CD without BIPSS if they met the criteria described in the consensus statement^7^. These patients were excluded from the current study because of lack of BIPSS results. Given the situation, the positive predictive value of the combined pituitary dMRI and HDDST will be higher with the enrollment of these patients. Also, some patients with incomplete medical records or conventional pituitary MRI instead of dMRI were excluded.

In summary, the combined pituitary dMRI and HDDST is non-invasive and cost-effective in the differential diagnosis of CS. It yields high positive predictive value and high accuracy of tumor lateralization. ACTH-dependent CS patients with both positive findings in pituitary dMRI and HDDST need no further invasive evaluation to establish the definite diagnosis of CD. BIPSS will improve the diagnosis accuracy when negative findings found in either pituitary dMRI or HDDST.
